# Measures of endothelial dysfunction predict response to cardiac resynchronisation therapy

**DOI:** 10.1136/openhrt-2015-000391

**Published:** 2016-06-07

**Authors:** David R Warriner, Patricia Lawford, Paul J Sheridan

**Affiliations:** 1 Medical Physics Group, Department of Cardiovascular Science, University of Sheffield, Sheffield, UK; 2 Department of Cardiology, Northern General Hospital, Sheffield Teaching Hospitals NHS Trust, Sheffield, UK

**Keywords:** HEART FAILURE, Flow Mediated Dilation

## Abstract

**Objectives:**

Cardiac resynchronisation therapy (CRT) improves morbidity and mortality in heart failure (HF). Impaired endothelial function, as measured by flow-mediated dilation (FMD) is associated with increased morbidity and mortality in HF and may help to differentiate responders from non-responders.

**Methods:**

19 patients were recruited, comprising 94% men, mean age 69±8 years, New York Heart Association functional classes II–IV, QRSd 161±21 ms and mean left ventricular ejection fraction 26±8%. Markers of response and FMD were measured at baseline, 6 and 12 months following CRT.

**Results:**

14 patients were responders to CRT. Responders had significant improvements in VO_2_ (12.6±1.7 to 14.7±1.5 mL/kg/min, p<0.05), quality of life score (44.4±22.9–24.1±21.3, p<0.01), left ventricular end diastolic volume (201.5±72.5 mL–121.3±72.0 mL, p<0.01) and 6-min walk distance (374.0±112.8 m at baseline to 418.1±105.3 m, p<0.05). Baseline FMD in responders was 2.9±1.9% and 7.4±3.73% in non-responders (p<0.05).

**Conclusions:**

Response to CRT at 6 and 12 months is predicted by baseline FMD. This study confirms that FMD identifies responders to CRT, due to endothelium-dependent mechanisms alone.

**Key questions**

**What is already known about this subject?**

- Response to cardiac resynchronisation therapy (CRT) is variable, and current guidelines do not adequately identify responders.

**What does this study add?**

- This study demonstrates measures of endothelial function, specifically flow-mediated dilation (FMD), accurately predict response to CRT at 6 and 12 months.

**How might this impact on clinical practice?**

- FMD could be used to identify potential responders at baseline to CRT.

## Introduction

In cardiovascular diseases, such as heart failure (HF), impairment of peripheral vascular endothelial function is common and can differentiate patients in terms of aetiology, functional class and prognosis.^1–3^ It has been demonstrated that endothelial function improves following successful use of a treatment such as cardiac resynchronisation therapy (CRT) and, more importantly, that a specific measure of endothelial function, termed flow-mediated dilation (FMD), measured a priori, can assist in the selection of patients likely to benefit from CRT.^4^ ^5^ This finding is important, as while it is well known that only two-thirds of patients implanted with CRT derive clinical benefit, current guidelines for CRT implantation (NYHA functional classes II–IV, EF%<35% and QRSd>120 ms) do not adequately select responders. Similarly, it is well known that echocardiographic parameters of dyssynchrony do not predict response.^6^

Akar first demonstrated that those with impaired endothelial function at baseline were more likely to respond to CRT and an improvement in endothelial function at 3 months follow-up post-CRT implantation.^4^ This study sought to confirm these findings, but with notable key differences. First, using peak VO_2,_ in addition to 6-min walk distance (6MWD), as a marker of response; second, using echocardiography-guided CRT optimisation protocol for all patients; third, following-up patients at 6 and 12 months to capture later responders; fourth, investigate the role of non-endothelium-dependent mechanisms, specifically nitroglycerine-mediated dilation (NMD) and finally, possible confounding of patient variables, for example, pacing site, scar location that may also influence CRT response.

NMD is an endothelial independent measure of vascular function, specifically, the maximal dilatory response to an administered nitric oxide (NO) donor, such as glyceryl trinitrate (GTN), and changes in this metric could confound the results of FMD, which has not been investigated. The working hypothesis was that there would be a significant difference in four measures of response at 6 and 12 months, and this response could be predicted by measures of endothelial dysfunction at baseline, prior to CRT implantation.

## Materials and methods

### Ethics

This study was approved by the local National Health Service (NHS) health research authority (NRES number 10/H0802/71). The authors take full responsibility for the integrity of the data. All authors have read and agree to the manuscript as it is written. None had any conflict of interest regarding this study. The patients were screened for eligibility by a physician and a dedicated clinical research fellow. All patients gave fully informed written consent to take part in the project.

### Inclusion criteria

All patients due to receive CRT based on current clinical criteria at Sheffield Teaching Hospitals NHS Trust were considered for this study. That is, patients with an ejection fraction <35%, a QRSd>120 ms, NYHA functional classes II–IV, and optimal medical therapy. A total of 21 patients were recruited at baseline, but two patients did not complete follow-up, and therefore, are not included in subsequent analysis.

### Study design

Following patient recruitment, endothelial function was assessed by means of FMD and NMD measured at baseline and repeated at 12 months (±2 weeks) post-CRT implantation. At 6 weeks post-CRT implantation, patients were referred for routine echocardiography-guided CRT optimisation, using the iterative method to optimise atrioventricular delay and the aortic velocity time integral (VTI) method for the interventricular delay.

### Assessment of endothelial function

**Figure 1:**
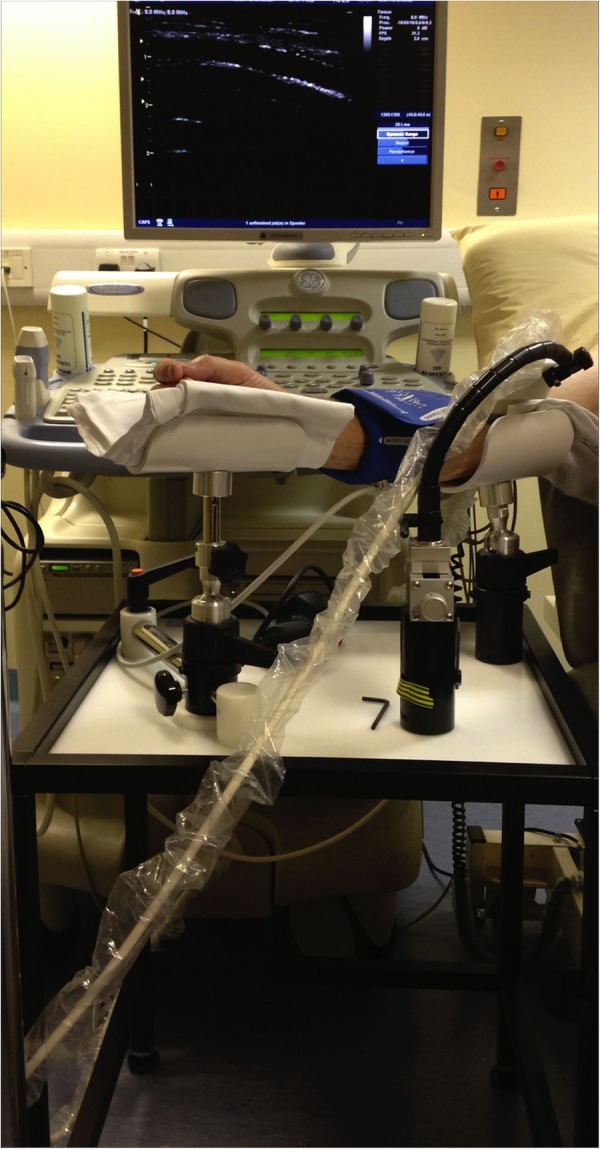
Custom built rig supporting the right upper arm and forearm, enabling the arm to be splinted in the anatomical position (elbow extended and forearm supinated), the ultrasound (USS) probe positioned proximal to the elbow and the sphygmomanometer cuff positioned distal to the elbow.

**Figure 2:**
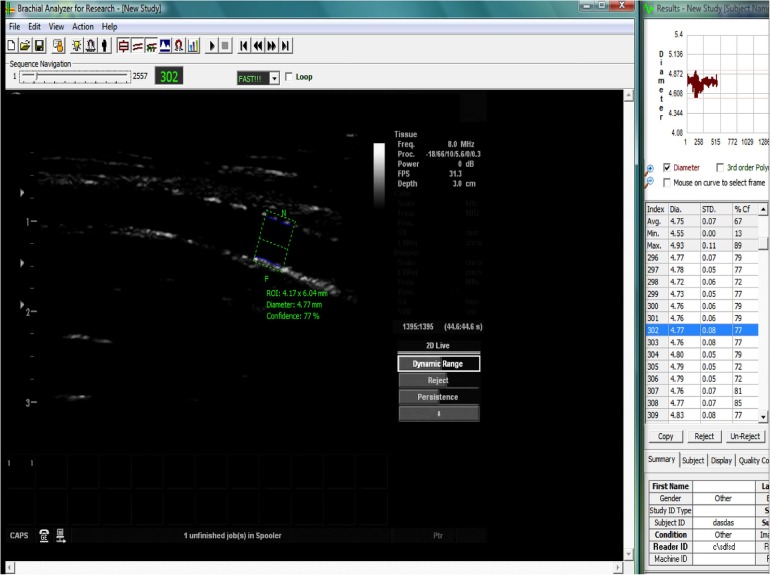
Screen shot of the MIA-IIc brachial analyser software recording the diameter of the brachial artery using B mode ultrasound, by identification of the lines of Pignoli in real time (green box=region of interest, blue lines=endothelium tracker).

Assessment of FMD was performed by a blinded investigator in accordance with the 2011 guidelines.^7^ The diameter of the brachial artery was measured (proximal to the elbow) for 1 min, to obtain a baseline measure, then a standard sphygmomanometer cuff was used over the forearm and inflated to >30 mm Hg above systolic blood pressure to occlude the forearm arteries. Following cuff deflation the brachial artery diameter was measured for a further 3 min. The artery was imaged using a custom-built rig (figure 1), comprising a Vivid 7 ultrasound machine (GE Healthcare, New Jersey, USA), and 2D Doppler probe with 8 MHz linear array. Images were relayed to a laptop (figure 2) using Epiphan frame-grabber device (Epiphan Systems Inc, Ottawa, California, USA) and analysed off-line using Medical imaging applications automated brachial analyser (MIA-llc, Iowa, USA). FMD was calculated as the percentage change in diameter from baseline.

**Figure 3:**
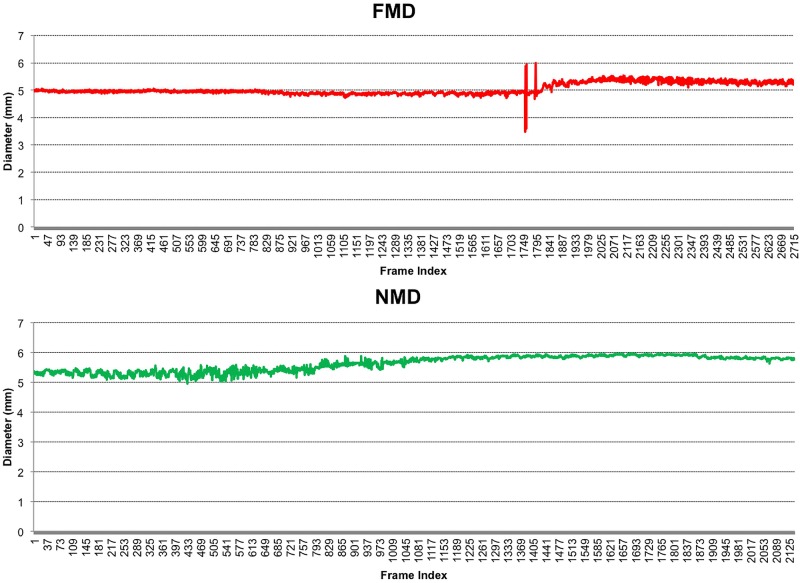
Demonstrating changes in brachial artery diameter, with FMD transient response following cuff deflation at 5 min (frame ∼1800) and sustained NMD response following administration of sublingual GTN (frame ∼600), recording at a rate of 5 frames per second.

Following a period of 20 min rest after the FMD, the same process was repeated but without the sphygmanometer in order to measure NMD. The patient was given 800 µg of GTN sublingually, the brachial artery imaged for a further 6 min, and the peak diameter recorded. NMD was calculated as the percentage change in diameter from baseline (figure 3).

### Assessment of response to CRT

Investigators assessing the clinical response were blinded to the FMD results and vice versa. A positive response to CRT was defined as that demonstrating an improvement in all four areas, a >1 mL/kg/min increase in peak VO_2_, a >10% reduction in left ventricular end-diastolic volume (LVEDV), a >10% reduction in symptoms as measured by the Minnesota living with heart failure questionnaire (MLWHFQ) and a >10% increase in 6MWD.

Peak VO_2_ was measured by a senior pulmonary physiologist during a cardiopulmonary exercise test using a ramp protocol on a static bicycle ergometer.

LVEDV was calculated by the modified Simpson's rule of stack discs using a 2D Vivid 7 echocardiography machine (GE Healthcare, New Jersey, USA) to image the apical two-chamber and four-chamber views. The images were assessed by experienced research sonographers.

The MLWHFQ, a validated and well-tested quality of life (QoL) questionnaire, specific for HF patients, and assessing both the psychological and physiological domains, was administered by the investigator.

The 6-min walk test measures the distance walked unaided, at a normal pace on a flat, hard, even surface in 6 min, termed the 6MWD.

### Statistics

Statistical analysis was performed using SPSS software (V.21.0, SPSS Inc, Chicago, Illinois, USA). Assessment of data for normality was carried out using Shapiro-Wilks test. Parametric data are given in terms of the mean±SD. Comparison of data within groups was performed using a paired two-tailed Student's t-test, and between groups using a one way ANOVA with repeated measures. Correlations were analysed with the Pearson's product coefficient. Nominal data was analysed using a two-tailed Fisher's exact.

p Values of <0.05 were considered significant. Simple linear regression was used to study the association between baseline FMD and baseline 6MWD, peak VO_2_, QRS duration, MLWHFQ and left ventricular (LV) volumes.

## Results

Baseline patient characteristics are summarised in table 1. As can be seen, all patients had similar levels of LV impairment and QRSd prolongation at baseline. There were no significant differences between the baseline measures of response, such as LVEDV, peak VO_2_, MLWHFQ or 6WMD.

**Table 1:** Baseline characteristics of responders and non-responders

	Responders (N=14)	Non-responders (N=5)	p Value
Demographics			
Age (years)	68.7±9.1	71.8±4.2	0.53
Male	13 (93%)	5 (100%)	0.50
Ischaemic heart disease	8 (57%)	3 (60%)	0.52
Hypercholesterolaemia	10 (72%)	4 (80%)	0.49
Hypertension	7 (50%)	2 (40%)	0.53
Chronic kidney disease	4 (29%)	4 (80%)	0.20
Diabetes	5 (36%)	1 (20%)	0.49
ECG/echocardiography			
QRSd (ms)	163.0±22.8	155.5±20.9	0.55
LVEDV (mL)	201.5±72.5	159.0±80.8	0.37
LVESV (mL)	156.5±57.3	116.4±68.2	0.23
SV (mL)	45.0±19.7	39.6±20.3	0.30
EF (%)	25.6±8.0	25.8±8.5	0.44
Medications (%)			
ACE-I/ARB	13 (93)	5 (100)	0.24
β-Blocker	12 (86)	5 (100)	0.90
Loop diuretic	14 (100)	5 (100)	0.36
MRA	9 (64)	4 (80)	0.93
Brachial diameter (mm)			
Baseline	4.43±0.67	4.40±0.63	0.94
6 months	4.47±0.54	4.50±0.37	0.94
12 months	4.56±0.57	4.71±0.36	0.56

ACE-I, ACE inhibitor; ARB, angiotensin receptor blocker; LVEDV, left ventricular end diastolic volume; LVESV, left ventricular end systolic volume; MRA, mineralocorticoid receptor antagonist; QRSd, QRS duration; SV, stroke volume.

### Response to CRT

**Figure 4:**
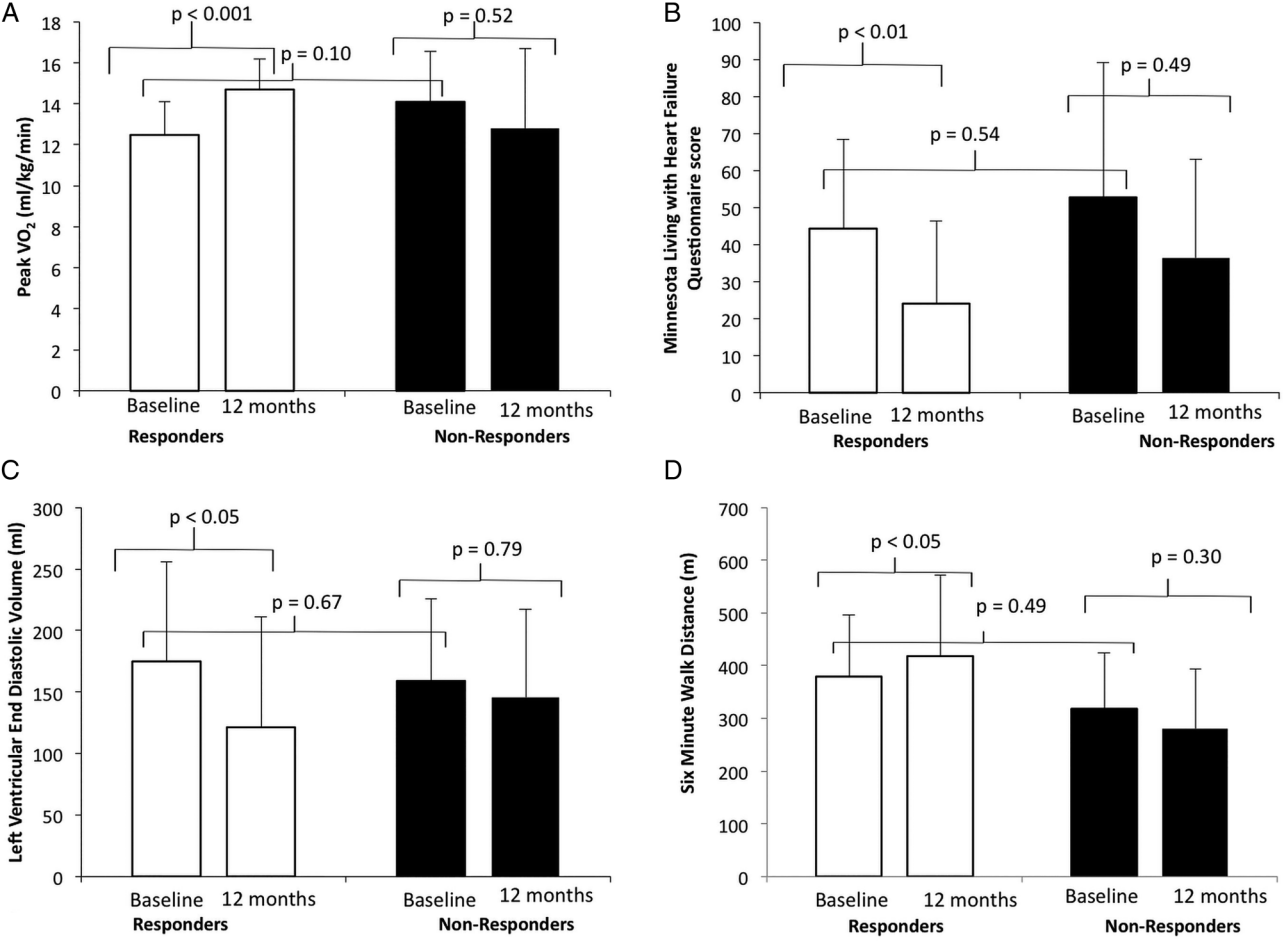
Response to cardiac resynchronisation therapy (CRT) (responders in white bars and non-responders in black bars). (A) Changes in peak VO_2_. (B) Changes in the Minnesota Living with Heart Failure Questionnaire (MLWHFQ) score. (C) Changes in Left Ventricular End Diastolic Volume (LVEDV) (D) Changes in 6-min walk distance (6MWD).

According to the four independent criteria specified a priori, a positive response to CRT was observed in 14 of the 19 patients during follow-up (table 2 and figure 4). Responders had significant improvements in cardiorespiratory fitness, symptoms and cardiac function. Specifically, at 12 months, responders had significant improvements in VO_2_ (12.6±1.7 to 14.7±1.5 mL/kg/min, p<0.05), QoL score (44.4±22.9 to 24.1±21.3, p<0.01), LVEDV (201.5±72.5 to 121.3±72.0 mL, p<0.01) and 6MWD (374.0±112.8 m at baseline to 418.1±105.3 m, p<0.05). The non-responders failed to demonstrate a statistically significant improvement in any of these four criteria at 6 or 12 months.

**Table 2:** Markers of response at baseline, 6 and 12 months

Markers of response	Responders	Non-responders	p Value
Baseline			
6MWD (m)	374.0±112.8	337.0±144.7	0.23
LVEDV (mL)	201.5±72.5	159.0±80.8	0.37
MLWHFQ	44.4±22.9	52.8±22.7	0.33
Peak VO_2_ (mL/kg/min)	12.5±1.6	13.9±2.7	0.25
6 months			
6MWD (m)	391.0±108.1	337.0±144.7	N/A
LVEDV (mL)	157.0±77.3	172.4±126.2	N/A
MLWHFQ	24.4±19.1	37.0±20.6	N/A
Peak VO_2_ (mL/kg/min)	14.1±2.7	11.5±4.1	N/A
12 months			
6MWD (m)	418.1±105.3	279.6±113.8	N/A
LVEDV (mL)	121.3±72.0	145.6±88.8	N/A
MLWHFQ	24.1±21.3	36.4±26.7	N/A
Peak VO_2_ (mL/kg/min)	14.7±1.5	12.8±3.9	N/A

6MWD, 6-minute walk distance; LVEDV, left ventricular end diastolic volume; MLWHFQ, =Minnesota living with heart failure questionnaire.

There were no significant differences during follow-up in β-blocker or ACE-I/ARB dose, but there was, however, a significant reduction in loop diuretic requirement of the responders at 12 months (83.1±24.9 to 62.2±37.6 mg, p<0.05), whereas this increased, albeit non-significantly in non-responders (70.0±39.3 to 100.0±49.0 mg, p=0.12).

In terms of LV reverse remodelling, there was a significant reduction in left ventricular end systolic volume (LVESV) in responders, at 12 months (156.5±57.3 to 127.0±39.4 mL, p<0.01), but this was not seen in non-responders (116.4±68.2 to 109.6±77.8 mL, p=0.49).

Finally, there were no clinical events during the 12 months follow-up, such as hospitalisation or death.

### Flow-mediated dilation

**Figure 5:**
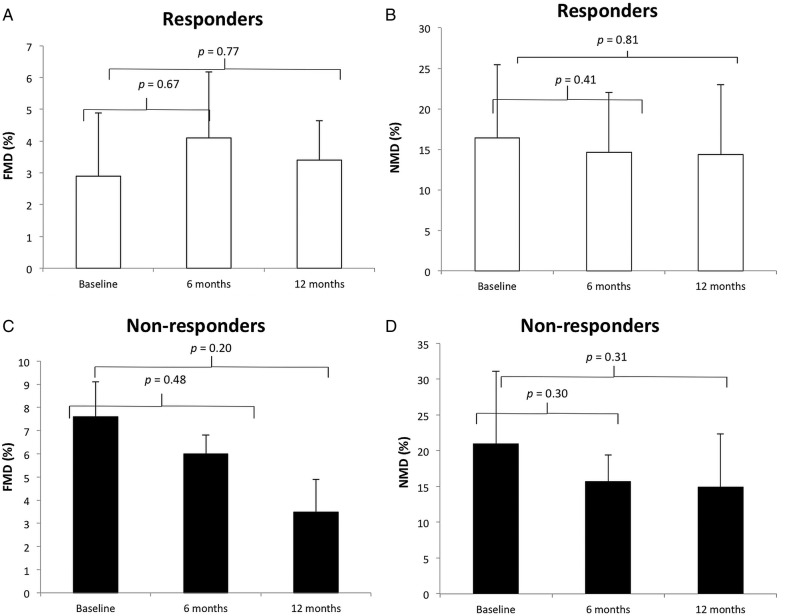
Endothelial function at baseline and follow-up (responders in white bars and non-responders in black). (A) Changes in flow-mediated dilation (FMD) following cardiac resynchronisation therapy (CRT) in responders, there were no significant differences at 6 or 12 months. (B) Changes in nitroglycerine-mediated dilation (NMD) following CRT in responders, there were no significant differences at 6 or 12 months. (C) Changes in flow-mediated dilation (FMD) following CRT in non-responders, there were no significant differences at 6 or 12 months. (D) Changes in nitroglycerine-mediated dilation (NMD) following CRT in non-responders, there were no significant differences at 6 or 12 months.

**Figure 6:**
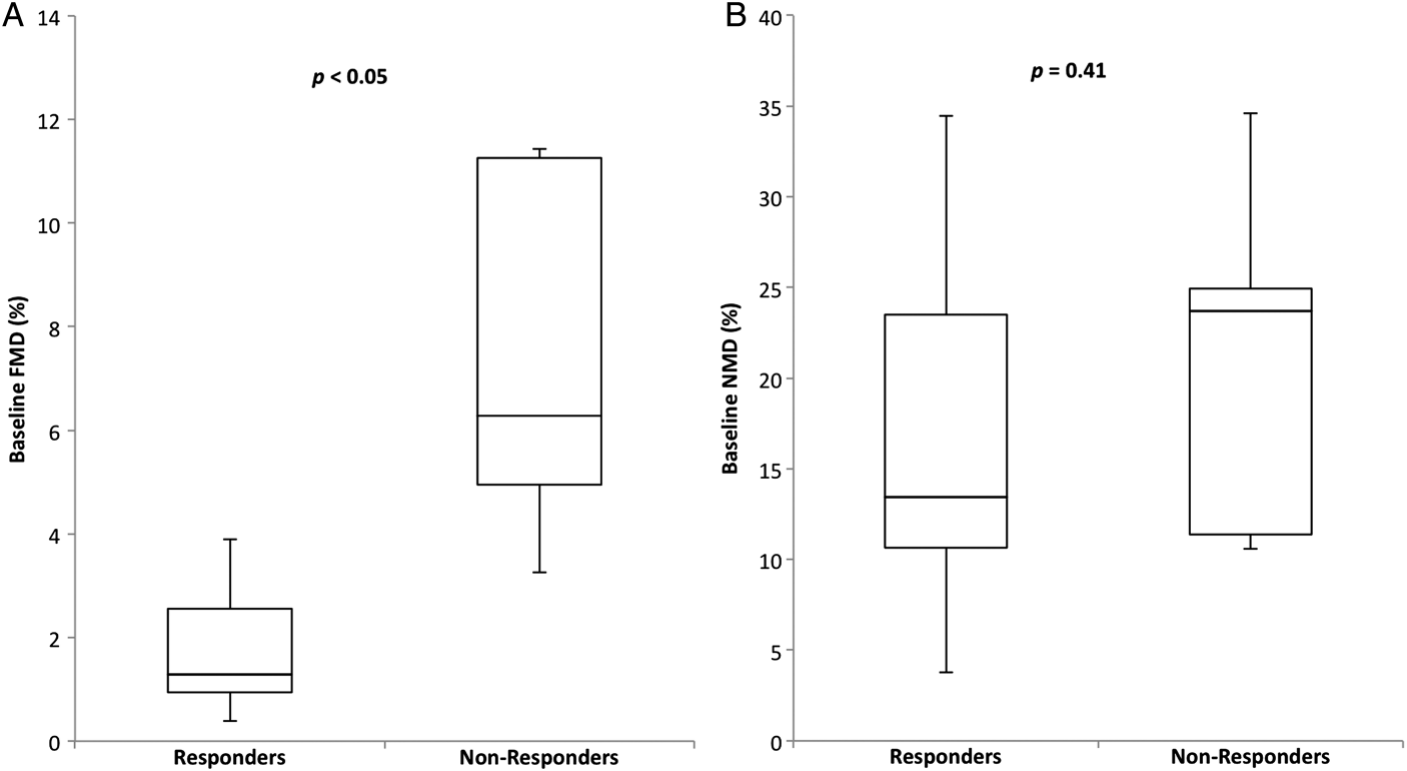
Box plots showing the distribution of baseline brachial artery flow-mediated dilation (FMD) and nitroglycerine-mediated dilation (NMD). Responders to cardiac resynchronisation therapy (CRT) demonstrated impaired endothelial function at baseline that was significantly lower than baseline in non-responders.

Baseline FMD in responders was 2.9±1.9% and 7.4±3.73% in non-responders (p<0.05, figures 5A, C and 6A). There was no difference in the resting diameter of the brachial artery measured prior to cuff inflation between baseline, 6 and 12 months in all 19 patients (table 1) ruling out the possibility that changes in the tone, or diameter, of the resting brachial artery were responsible for the changes in FMD. During follow-up, there was an improvement in endothelial function as measured by FMD in the responder group (figure 5A), and a deterioration in the non-responder group (figure 5C), but these changes did not reach statistical significance.

### Nitroglycerine-mediated dilation

There was no significant difference in baseline NMD between responders and non-responders (16.7±9.41% and 21.0±10.1, respectively, p=0.41, figure 6B). During follow-up, there was no significant change in NMD, in either the responders or non-responders (figure 5B, D).

### FMD and response to CRT

**Figure 7:**
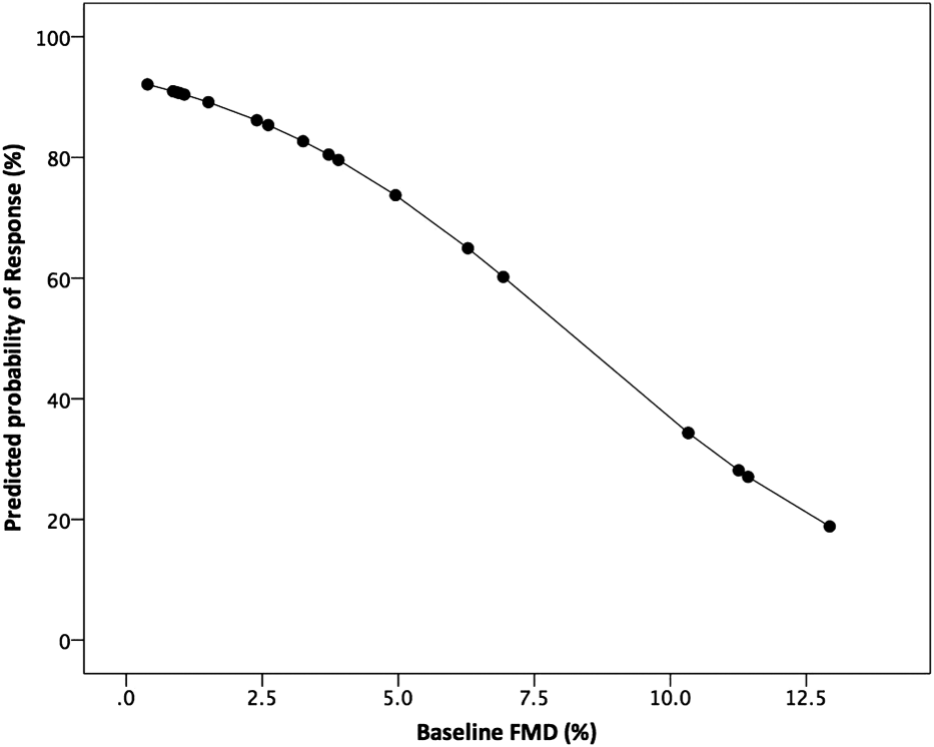
Logistic regression analysis model of the probability of response to cardiac resynchronisation therapy (CRT) and baseline endothelial function using flow mediated dilation (FMD). For every 1% reduction in baseline FMD, there was an approximate 8% increase in the likelihood of CRT response.

There was no correlation between baseline FMD and markers of response, nor any correlation between improvement in FMD and improvement in markers of response. Using logistic regression analysis, baseline FMD independently predicts likelihood of response to CRT. This demonstrates that for every 1% reduction in baseline FMD the likelihood of CRT increased by 8% (figure 7).

### Other factors affecting CRT response

As can be seen from table 3, regardless of response, the majority of patients had an intraventricular conduction delay of left bundle block morphology, pacing leads placed in the right atrial appendage, right ventricular apex and the lateral or posterior vein, and finally scar, if present, in the anterior wall or septum. All 19 patients were receiving over 95% biventricular pacing during follow-up, verified with departmental pacing checks and remote monitoring.

**Table 3:** Other factors affecting CRT response

Other factors		Responders (%)	Non-responders (%)	p Value
Bundle branch block	Left	13 (93)	5 (100)	0.56
CRT device	P only	5 (36)	3 (60)	0.37
	Right atrium			
Lead position	Appendage	9 (65)	4 (80)	0.65
	Right ventricle			
	Apex	14 (100)	5 (100)	0.99
	Left ventricle			
	Lateral vein	10 (72)	4 (80)	N/A
	Middle vein	1 (7)	0 (0)	
	Posterior vein	2 (14)	1 (20)	
	Epicardial	1 (7)	0 (0)	
Presence of scar	Anterior wall	4 (29)	2 (60)	0.91
	Apex	1 (7)	0 (0)	
	Lateral wall	1 (7)	0 (0)	
	Inferior wall	1 (7)	0 (0)	
	Septum	1 (7)	1 (20)	

## Discussion

This study demonstrates that FMD predicts clinical response to CRT, is lower at baseline in non-responders, but that FMD predicts response at 6 and 12 months. There were no significant clinical differences between the responders and non-responders at baseline; all met the current guidelines for CRT implantation. If FMD is used at baseline to discriminate responders from non-responders, then non-responders may not be exposed to potential harms from a device they will ultimately not benefit from.

It is uncertain why responders at baseline should have lower FMD, as while patients with ischaemic cardiomyopathy often have worse endothelial function, it is patients with non-ischaemic cardiomyopathy who typically demonstrate a greater response to CRT.^3^ ^8^ It is possible that differences in endothelial function, are a result of ‘altered haemodynamics, peripheral shear stress, cardiac loading conditions and neurohormonal activation’.^4^ Indeed, there was no significant difference in HF aetiology between the groups to otherwise account for this difference in FMD at baseline. During follow-up, endothelial function, as measured by FMD, appeared to deteriorate in non-responders and improved in FMD in responders, as found previously, although this was not significant in either case.^4^

There are a number of key procedural differences between the author's work and the previous studies, including longer follow-up, markers of response, use of optimisation, use of NMD and investigation of possible confounding variables. The present study followed-up patients for 12 months. This is consistent with large randomised CRT trials, such as MUSTIC, MIRACLE and CONTAK, and demonstrates that FMD predicts medium, not just short-term response to CRT.^9–11^ As was previously predicted, but not investigated, measures of response to CRT based on brachial artery reactivity are entirely endothelially mediated, with this study showing no significant differences in NMD before or after CRT.^4^ Unlike previous work, there was no improvement in FMD following CRT, indeed there was deterioration in both FMD and NMD, suggesting a continuing decline in endothelial function, but the study was not powered to detect these changes. Cardiac function, cardiorespiratory fitness and symptoms were all measured at baseline and during follow-up. Peak VO_2_ is considered the gold standard measure of cardiorespiratory function, and is used in conjunction with a significant improvement in the other four markers of response, gave a more robust categorisation of response, and reducing the element of chance, rather than simply relying on one metric alone. As seen in table 3, there was also no significant difference between other possible extraneous factors influencing CRT response, such as bundle branch morphology, presence of scar or lead placement, for example, not investigated previously. However, larger studies including scar location, lead placement and type of device, will be required in order to rule out such variables as influencing either CRT response or indeed, FMD.

Endothelial dysfunction is important in cardiovascular disease, and reflecting ‘increased arterial stiffness and reduced compliance increase ventricular afterload and left ventricular end-diastolic stress, and enhance dilation and failure’, and ‘impaired function of the large epicardial coronary arteries, as well as the coronary microcirculation, which may cause or contribute to myocardial ischemia’.^2^ Thus, FMD is not simply an arbitrary number, but rather is a measure of the fitness of the patient's endothelium, an important ‘organ’ which is often overlooked. Indeed, exercise training can further improve endothelial function following successful CRT implantation and clinical response.^12^

Larger trials are needed to confirm the power of FMD to predict response to CRT before it can be used as part of a randomised controlled trial and a threshold for FMD predicting response is determined. Further work will be needed in order to investigate the role of HF aetiology in determining FMD and response to CRT, for example, ischaemic versus non-ischaemic HF, the role of comorbidity such as chronic kidney disease or pulmonary hypertension, and also QRS morphology, for example, left versus right bundle branch block.

This was a small prospective study, but it was adequately powered to detect differences in baseline FMD among the two groups.^13^ Limitations include a slightly smaller response rate in the present study than in larger randomised controlled trials. This can be attributed to a small sample size and multimodal classification of response a priori. There was only one woman and no ethnic minorities in this cohort, so it is difficult to translate the results to such populations. Defining response to CRT is problematic and subjective, for this reason the authors decided that any responder must demonstrate improvement in four different markers at prespecified thresholds, for example, peak VO_2_, 6MWD, LVEDV, MLWHFQ, widely used in the literature.^14^^–^20 This was to ensure, as much as possible, that any response was true and not due to random variation, particularly in such a small patient population. While, the non-responder group demonstrated an improvement in QoL, as measured by the MLWHFQ score, this was not significant and likely to be due to a combination of chance and placebo effect.

## Conclusions

In the current study, FMD predicts the response to CRT at 6 and 12 months. These results confirm previous work reporting that measures of endothelial dysfunction identifies response to CRT at 3 months, and by contrast, NMD is not a predictor of response to, nor significantly influenced by, CRT. Consideration needs to be made to using FMD in routine clinical practice in the selection of patients for CRT. But first, larger studies will be needed to confirm FMD's predictive power, and determine why patients with poorer endothelial function, at baseline, are more likely to respond to CRT.
